# A Novel Role for PECAM-1 (CD31) in Regulating Haematopoietic Progenitor Cell Compartmentalization between the Peripheral Blood and Bone Marrow

**DOI:** 10.1371/journal.pone.0002338

**Published:** 2008-06-04

**Authors:** Ewan A. Ross, Sylvie Freeman, Yan Zhao, Tarvinder S. Dhanjal, Emma J. Ross, Sian Lax, Zubair Ahmed, Tie Zheng Hou, Neena Kalia, Stuart Egginton, Gerard Nash, Steve P. Watson, Jon Frampton, Christopher D. Buckley

**Affiliations:** 1 Rheumatology Research Group, MRC Centre for Immune Regulation, University of Birmingham, Birminham, United Kingdom; 2 Division of Immunity and Infection, MRC Centre for Immune Regulation, University of Birmingham, Birmingham, United Kingdom; 3 Center for Cardiovascular Studies, University of Birmingham, Birmingham, United Kingdom; 4 Molecular Neuroscience Group, University of Birmingham, Birmingham, United Kingdom; 5 Department of Physiology, University of Birmingham, Birmingham, United Kingdom; Dartmouth Medical School, United States of America

## Abstract

Although the expression of PECAM-1 (CD31) on vascular and haematopoietic cells within the bone marrow microenvironment has been recognized for some time, its physiological role within this niche remains unexplored. In this study we show that PECAM-1 influences steady state hematopoietic stem cell (HSC) progenitor numbers in the peripheral blood but not the bone marrow compartment. PECAM-1^−/−^ mice have higher levels of HSC progenitors in the blood compared to their littermate controls. We show that PECAM-1 is required on both progenitors and bone marrow vascular cells in order for efficient transition between the blood and bone marrow to occur. We have identified key roles for PECAM-1 in both the regulation of HSC migration to the chemokine CXCL12, as well as maintaining levels of the matrix degrading enzyme MMP-9 in the bone marrow vascular niche. Using intravital microscopy and adoptive transfer of either wild type (WT) or PECAM-1^−/−^ bone marrow precursors, we demonstrate that the increase in HSC progenitors in the blood is due in part to a reduced ability to migrate from blood to the bone marrow vascular niche. These findings suggest a novel role for PECAM-1 as a regulator of resting homeostatic progenitor cell numbers in the blood

## Introduction

The bi-directional movement of HSCs between the vascular niche and the circulation is an area of intense study. Alterations in integrins, chemokine receptors and the activation of proteases have all been demonstrated to be required for HSC mobilization and subsequent engraftment to occur efficiently [1], [2] Of particular importance is the localised concentration of CXCL12 (SDF-1α) in the bone marrow as this is the principal chemokine to which HSCs respond[3]. This chemokine not only acts as a chemoattractant for HSCs, but ligation of its receptor, CXCR4, induces integrin upregulation and promotes HSC survival[4]–[7]. Modulation of the CXCR4/CXCL12 interaction on HSCs has been shown to directly affect HSC migration and function[8].

Platelet Endothelial Cell Adhesion Molecule-1 (PECAM-1/CD31) is a 130kDa glycoprotein and member of the immunoglobulin superfamily, which is expressed on many haematopoietic and endothelial cells[9]. PECAM-1 is expressed on the surface of various cells of the haematopoietic lineage[10]–[12], however the function of PECAM-1, if any, on haematopoietic progenitor cells and the bone marrow vasculature remains to be fully elucidated. It has been observed that expression of PECAM-1 decreases following the emigration of CD34^+^ cells and monocytes[13] from the bone marrow to the blood[13], [14]. The expression of PECAM-1 on CD34^+^ cells, like that on endothelial cells, has been demonstrated to provide an anti-apoptotic signal[15], [16], with ligation of PECAM-1 enhancing VLA-4 mediated adhesion[17], and blocking antibodies to PECAM-1 reducing transendothelial migration[18], [19].

We and others have recently discovered a novel role for PECAM-1 in regulating CXCR4 dependent megakaryocyte migration within the bone marrow vascular niche[20], [21]. The expression of PECAM-1 on HSCs and the bone marrow vasculature, together with the well established role for PECAM-1 in regulating cell adhesion, migration and activation in cells of haematopoietic origin prompted us to examine whether PECAM-1 could regulate HSC function in the bone marrow vascular niche.

We observed that PECAM-1 regulates steady state progenitor cell numbers in the peripheral blood but not the bone marrow compartment. PECAM-1^−/−^ mice exhibited an increase in the number of circulating progenitor cells in the resting state, compared to littermate wild type control mice. Using intra-vital microscopy, we found that loss of PECAM-1 on either bone marrow progenitors or vascular cells resulted in the failure of cells to migrate across the bone marrow vasculature leading to their accumulation within the peripheral circulation. We have identified an intrinsic migration defect of HSCs to CXCL12 as well as increased levels of MMP-9 in the bone marrow of PECAM-1^−/−^ mice, suggesting that factors intrinsic to both the HSC and bone marrow vascular niche account for the increase in circulating progenitor numbers in the blood of PECAM-1^−/−^ mice.

Our results suggest that PECAM-1 plays an important role in integrating CXCL12 dependent signals involved in HSC accumulation within the bone marrow, such that loss of PECAM-1 prevents effective bidirectional movement of HSCs between the blood and bone marrow compartments.

## Materials and Methods

### Mice and bone marrow collection

Age and sex matched wild type (WT) and PECAM-1^−/−^ littermates on the C57BL6J background were bred from heterozygotes. All mice were maintained at the Biomedical services unit at the University of Birmingham, according to Home Office regulations. Bone marrow cells were flushed from femurs and tibias of 6–9 week old mice, and resuspended in ACK lysis buffer (0.15M NH_4_Cl; 1 mM KHCO_3_; 0.1 mM Na_2_EDTA pH7.3) to lyse mature erythrocytes before use in subsequent assays.

### Flow Cytometric Analysis

Bone marrow cell populations were quantified and phenotyped using a CyAn ADP flow cytometer (Dako, Ely, UK). The following monoclonal antibodies were used: anti-mouse lineage panel-APC, anti-c-Kit-RPE, anti-Sca-1-PE/Cy7, anti-CD127-Pacific Blue (all BD Biosciences), anti-CD34-FITC, anti-CD16/CD32-PE/Cy5 and biotinylated anti-CD135 (all eBioscience, Insight Biotechnology, UK). Biotinylated antibodies were visualised with streptavidin-APC/Cy7 (BD Bioscience). Chemokine receptor and integrin expression was demonstrated using biotinylated anti-CXCR4, anti-CD49d or FITC labelled anti-CD18, CD29 and CD61 antibodies (all BD Biosciences). The HSC population was defined as c-Kit^+^, Sca-1^+^ but lineage^−^ (KSL population), and at least 1×10^6^ total bone marrow events were analysed per sample . KSL cells were purified by double FACS sorting using a high speed cell sorter (Dako, Ely, UK).

### Cell Migration Assays

Transwell chamber assays were performed in transwell buffer (RPMI supplemented with 2 mM glutamine, 100units/ml penicillin, 100 µg/ml streptomycin and 0.5% Fraction V BSA; all Sigma-Aldrich, UK). Recombinant murine CXCL12 (Peprotech EC, UK) was added to the bottom chamber at 200–500 ng/ml. Cell suspensions were isolated and resuspended at 2×10^6^/ml in transwell buffer, before adding 100 µl to the top chamber. Assays ran for 4 hours before collecting and quantifying the cells migrated into the bottom chamber using a fixed volume count flow cytometry protocol (Epics XL, Coulter). PI was added to each tube to discriminate dead cells and debris.

Dunn chamber assays were performed using HSCs purified by immunomagnetic bead selection. Whole bone marrow was firstly negatively depleted with lineage specific beads (Lineage Cell Depletion Kit, Miltenyi Biotech) according to the manufacturer's instructions. The resulting cells were then positively selected using CD117 Microbeads (Miltenyi Biotech) and rested overnight in StemCell Pro media (supplemented with 2.6% serum replacement, 2 mM glutamine, 100 units/ml penicillin, 100 µg/ml streptomycin), and containing factors to promote survival and inhibit differentiation (50 ng/ml recombinant SCF and 10 ng/ml recombinant Flt-3L; Peprotech EC, UK). Dunn chambers were performed as previously described[20], [22], using 2×10^5^ cells per assay.

### G-CSF Induced CFC Mobilisation

The induced mobilisation of bone marrow HSCs was performed using a protocol adapted from Petit *et al.* [23]. Briefly, mice received a daily subcutaneous injection of G-CSF (Neupogen, Amgen Inc, USA; 300 µg/kg in 100 µl PBS) for 4 consecutive days and were sacrificed 6 hours after the last injection. Peripheral blood was collected by cardiac puncture into heparinised tubes, and leg bones removed. The quantification of CFCs in bone marrow and peripheral blood was performed using MethoCult GF media (M3434, Stemcell Technologies, UK), according to the manufacturers protocol. Two independent researchers counted all colonies on duplicate plates for each condition tested at days 7 and 10.

### CXCL12 ELISA

CXCL12 levels in bone marrow were measured by flushing the femurs and tibias from one mouse with 500 µls of PBS. Cells were centrifuged, supernatants aliquoted and frozen at −80°C. ELISAs were performed according to the protocol published by Petit *et al.* [23]. Briefly, wells of a 96-well plate were coated overnight with 50 µls of anti-CXCL12 antibody (MAb 79018, R&D Systems, UK) at a concentration of 2 µg/ml in bicarbonate buffer (15 mM Na_2_CO_3_; 35 mM NaHCO_3_ pH9.6). Wells were washed 3 times, blocked for 1 hour with PBS/1%BSA/5% Sucrose at room temperature, rewashed 3 times and 50 µl of sample in duplicate wells added for 2 hours at room temperature. Recombinant murine CXCL12 (Peprotech EC) was used to generate a standard curve. Wells were washed 3 times and 50 µls of anti-CXCL12-biotin capture antibody (MAb BAF310, R&D Systems, UK) at 250 ng/ml for 2 hours at room temperature. After 3 washes, 50 µl of streptavidin-HRP was added for 20 mins at room temperature, washed for a further 3 times, and incubated for 20–30 mins with 50 µl of TMB substrate solution (Sigma-Aldrich, UK). Reactions were stopped with 50 µl of 2M H_2_SO_4_ and wells read at 450 nm on an optical plate reader. All samples analysed were quantified for protein concentration and results expressed as ng/ml CXCL12 per mg/ml protein in solution.

### Indirect Immunofluorescence staining and confocal microscopy

Cryosections (8 µm) of mouse femur were fixed in acetone and blocked with 5% mouse and 5% goat serum in PBS. Sections were stained with the following antibodies: From Ebioscience anti-endoglin-biotin (clone MJ7/18) and anti-VCAM-1-FITC (clone 429). Anti-CXCL12 (clone 79018) was from R&D Systems. Secondary reagents were anti-FITC-Alexa 488 (Molecular Probes), goat anti-mouse IgG1-TRITC (Southern Biotech) and Streptavidin-Cy5 (Jackson Immuno Research). Cell nuclei were visualised using Hoescht 33258. A Zeiss confocal LSM 510 microscope (Zeiss, Germany) was used to visualize staining with images captured and processed using the Zeiss LSM Image Examiner software (Zeiss).

### Intravital Imaging of the Bone Marrow Microcirculation

Intravital observations of the bone marrow were made using the skull window preparation previously described by Mazo and colleagues[24]. All procedures were undertaken with United Kingdom Home Office approval in accordance with the Animals (Scientific Procedures) Act of 1986 (Project Licence No: 40/2749). Anaesthesia was induced with an intraperitoneal injection ketamine (100 mg/kg Vetalar; Pharmacia and Upjohn Ltd, UK) and 2% xylazine (20 mg/kg; Millpledge Pharamaceuticals, UK). Cannulae were inserted in the trachea to facilitate spontaneous respiration and in the left carotid artery to provide a route for administration of additional anaesthesia as required. The carotid cannula also provided access for administration of fluorescently labelled albumin and cells. The scalp was incised in the midline and the frontoparietal skull was exposed carefully avoiding tissue damage to the area. A plastic ring was placed to maintain exposure of the incision and to allow application of sterile saline to prevent drying of the tissue. The animal was immobilised in a custom made stereotactic frame.

Animals were transferred to the stage of a motorized Olympus BX-61WI microscope equipped with a water immersion objective (Olympus UmplanFI 10x/0.30w). The microvessels were identified after administration of FITC-BSA (0.1 mg/ml, Sigma, UK). Following this, whole bone marrow cells were isolated from femurs as previously described, labelled with 5 µM CFSE (Molecular Probes, Invitrogen, Paisley, UK) and introduced systematically. Digital images were collected using a high capture rate Sensicam CCD camera (the Cooke Corporation, USA). A high performance GEN III image intensifier (Videoscope Int. Ltd., USA) incorporated between the microscope and camera amplified light 1000-fold, allowing visualization of low fluorescent intensities. Images were collected using Slidebook software (Intelligent Imaging Innovations, USA) and stored as permanent digital images for subsequent off line analysis. One pre-selected field of view was recorded for 5 mins after administration of bone marrow cells to allow quantification of rolling cells. To quantify adherent cells, a second recording of the entire exposed left frontoparietal area was captured one hour post-injection. Cells adherent to the microvessels for <30 seconds were considered adherent.

### Zymography

Bone marrow flushes were spun down to remove cell debris and quantified for protein content by BCA assay (Pierce, UK). 20 µg total protein was prepared in nondenaturing loading buffer and size fractionated in 10% SDS-polyacrylamide gel containing 1 mg/ml collagen. Gels were washed twice in 2.5% Triton X-100 and incubated overnight at 37°C in 10 mM Tris-HCl buffer, pH 7.5 containing 1.25% Triton X-100, 5 mM calcium chloride and 1 µM zinc chloride. Gels were fixed in 50% methanol and 10% acetic acid containing 0.25% Coomassie Blue for 2 hours, and the digested bands revealed by destaining with 25% methanol and 10% acetic acid solution. Gels were scanned and quantified as previously described[25].

### Statistical Analysis

All experiments were performed a minimum of three times and images shown are representative of one experiment performed. Data are shown as mean±S.D. and statistical analysis was performed using 2-tailed Student's *t* test. Probability values of *P* below 0.05 were considered to be statistically significant.

## Results

### Phenotypic analysis of HSC numbers and differentiation in the bone marrow of PECAM-1^−/−^ mice

**Figure 1:**
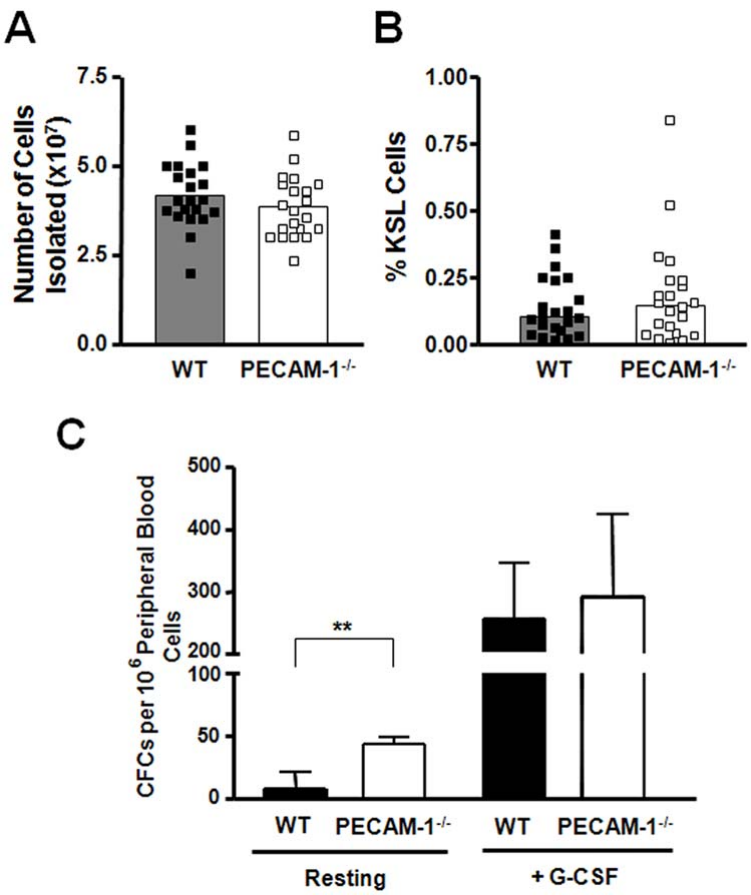
Analysis of haematopoietic progenitors in the bone marrow and peripheral blood. (A) There was no significant difference in total bone marrow cell numbers between PECAM-1^−/−^ and WT mice (n = 21). (B) The percentage of KSL cells in the bone marrow cellular pool was measured by flow cytometry, and no significant difference was observed (n = 22 pairs). Bars in (A) and (B) represent mean values, and in both cases no significant difference was observed. (C) The number of colony forming cells (CFCs) in peripheral blood of mice was determined using methylcellulose assays. Data shown is the mean±S.D. of 6 pairs of littermate mice, **p = 0.008. To measure the ability of PECAM-1^−/−^ HSCs to mobilise from the bone marrow, mice were treated with a daily injection of G-CSF. No significant difference in the number of CFCs released into the peripheral blood was found. (n = 6 pairs of littermates).

We first examined HSC cell numbers in the bone marrow of PECAM-1 deficient mice compared to age and sex matched littermate controls. Total bone marrow cell numbers were the same in bone marrow flushes from WT littermates and PECAM-1^−/−^ mice (Figure 1A). The number of HSCs, defined by KSL staining, in PECAM-1 deficient mice was not significantly different compared to WT littermate controls (WT 0.16%±0.14, KO 0.21%±0.2, n = 21) (Figure 1B). A similar observation was made using side population analysis[26] (data not shown). This suggests that bone marrow cell homeostasis is maintained upon PECAM-1 deletion.

We next examined whether there were differences in progenitor subsets in the bone marrow based on previously published phenotyping information[27]–[29]. Table 1 shows there were no significant differences in the numbers of the commonly described subsets of HSC progenitors in the WT and PECAM-1^−/−^, mice suggesting that loss of PECAM-1 does not drive differentiation into one particular haematopoietic lineage. Similar findings were observed using the recently described protocols to differentiate HSCs based on their expression of the SLAM family receptors CD48 and CD150[30] (data not shown).

**Table 1:** Flow cytometric analysis of haematopoietic progenitor subsets.

Subset	Phenotype	% Total BM
Dormant HSC (LT-HSC)	Lin^−^ CD34^−^ CD135^−^ Sca-1^+^ CD117^+^	**WT** 0.0096±0.0036 **KO** 0.0117±0.0047
Activated HSC (ST-HSC)	Lin^−^ CD34^+^ CD135^−^ Sca-1^+^ CD117^+^	**WT** 0.0224±0.0164 **KO** 0.0291±0.0273
MPP	Lin^−^ CD34^+^ CD135^+^ Sca-1^+^ CD117^+^	**WT** 0.0355±0.0228 **KO** 0.0291±0.0273
CLP	Lin^−^ CD127^+^ Sca-1^int^ CD117^int^	**WT** 0.014±0.019 **KO** 0.013±0.015
GMP	Lin^−^ CD127^−^ CD34^+^ CD16/32^+^ Sca-1^−^ CD117^+^	**WT** 0.081±0.054 **KO** 0.071±0.052
MEP	Lin^−^ CD127^−^ CD34^−^ CD16/32^−^ Sca-1^−^ CD117^+^	**WT** 0.201±0.154 **KO** 0.241±0.153
CMP	Lin^−^ CD127^−^ CD34^+^ CD16/32^low^ Sca-1^−^ CD117^+^	**WT** 0.100±0.087 **KO** 0.117±0.059

Bone marrow cells were isolated and subgroups of haematopoietic progenitor cells were identified by flow cytometry from 6–8 week old mice. Data shown is from at least 6 littermate pairs. *LT-HSC* Long-Term HSC; *ST-HSC* Short-Term HSC; *MPP* Multipotent Progenitor; *CLP* Common Lymphoid Progenitor; *CMP* Common Myeloid Progenitor; *GMP* Myelomonocytic Progenitor; *MEP* Megakaryocytic/Erythrocyte Progenitor. No significant difference between WT and PECAM-1^−/−^ mice was observed in any of the populations studied.

### Increased numbers of functional progenitor cells in the steady state circulation of PECAM-1^−/−^ mice

We next examined whether loss of PECAM-1 affected the accumulation of HSC progenitors in the peripheral blood, using colony forming assays (Figure 1C). We observed a significant increase in the number of CFCs in the blood of PECAM-1^−/−^ mice in steady state conditions compared to WT littermate controls. Following enforced mobilization with G-CSF there were no differences in either the number of CFCs in the blood (Figure 1C) or the percentage of KSL cells in the bone marrow in both WT and PECAM-1^−/−^ mice (data not shown), suggesting that loss of PECAM-1 selectively affects progenitor cells numbers in the steady state circulation, but does not affect their ability to be actively mobilized.

### PECAM-1^−/−^ HSC cells demonstrate impaired migration to CXCL12 (SDF-1α)

**Figure 2:**
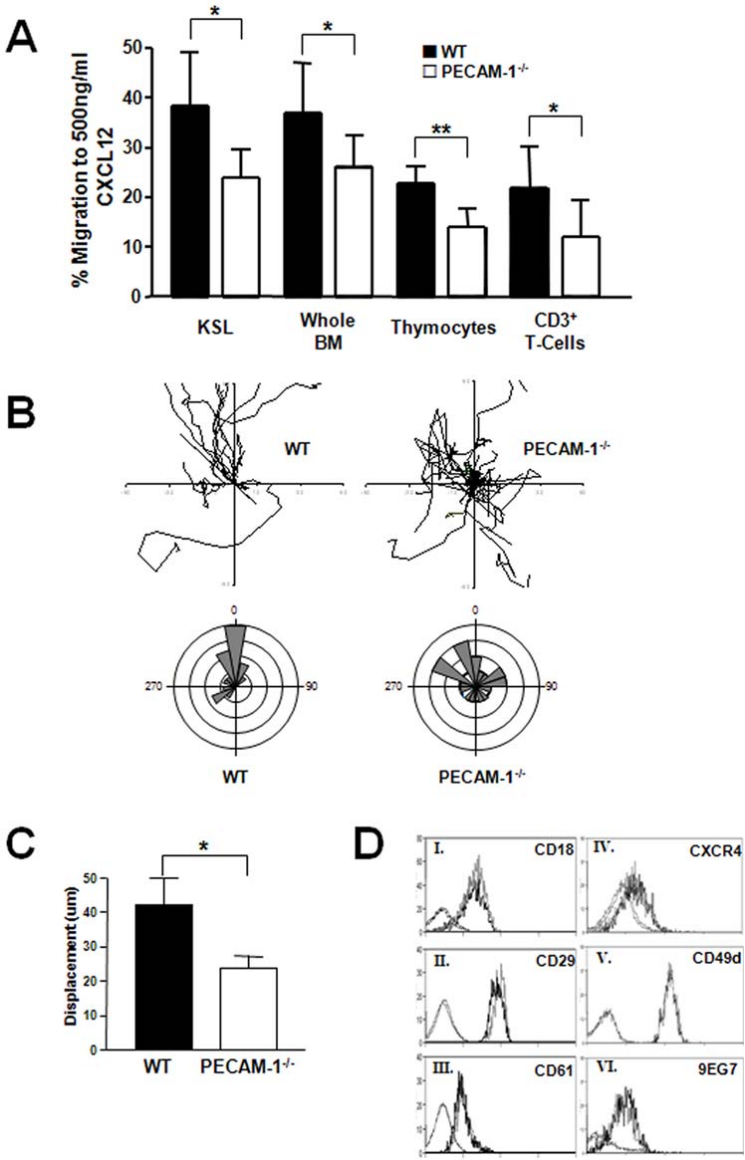
PECAM-1^−/−^ HSCs demonstrate defective cell migration towards a gradient of CXCL12. (A) 0.1∼1×10^6^ cells isolated from individual PECAM-1^−/−^ and littermate matched WT animals were placed in a transwell chamber and their ability to migrate to a gradient of CXCL12 was quantified from at least 3 pairs of mice; cell sorted KSLs (n = 3, p = 0.019), whole bone marrow (n = 7, p = 0.02), thymocytes (n = 4, p = 0.005) and purified CD3^+^ T cells (n = 7, p = 0.03). (B) Dunn chambers were used to trace the migration path over one hour of 20 lineage depleted, CD117^+^ progenitor cells. The intersection of the x- and y-axis was taken as the starting point of each cell path, and the source of CXCL12 was at the top. Circular histograms show the proportion of cells whose final position was located within each of 18 equal sectors (20°). (C) The net translocation distance (displacement from start to end point) of each cell was measured (mean±S.D.; *p>0.05). (D) Expression levels of surface proteins on KSL cells were quantified on whole bone marrow isolates by flow cytometry. Histograms I-VI are representative of at least four independent experiments, and no significant difference in staining was observed between PECAM-1^−/−^ (grey) and WT (black) littermate controls, except for CD29 (ii, n = 5, *p = 0.038). Faint histograms depict the appropriate isotype controls.

CXCL12 (SDF-1α) is critically involved in the regulation of HSC accumulation and migration, both within the bone marrow niche and between the bone marrow and the peripheral circulation[5]. We therefore examined the ability of HSCs from PECAM-1^−/−^ and littermate controls to migrate to CXCL12. Purified KSL cells were isolated from whole bone marrow by cell sorting (≥99% purity), and migration to CXCL12 assessed using a Transwell assay. PECAM-1^−/−^ KSL cells displayed a reduced capacity to migrate to CXCL12 compared to WT littermate controls (Figure 2A). A similar effect was observed using whole bone marrow extracts, isolated thymocytes and purified mature CD3^+^ splenic T cells suggesting that the altered migration response to CXCL12 is an intrinsic property of the PECAM-1^−/−^ HSCs, and that the defect persists throughout haematopoietic development in lymphocytes. Whole bone marrow cell preparations were also assayed using other chemotactic agents including MCP-1, KC, RANTES and IL-8, but no difference in the migratory capacity of WT and PECAM-1^−/−^ was observed to these chemotactic agents (data not shown). Thus the defect in migration of PECAM-1^−/−^ bone marrow cells appears to be specific to CXCL12.

These findings using static transwell assays were confirmed using Dunn chamber migration assays to assess the speed and direction of cell migration. Lineage negative, CD117^+^ progenitor cells were allowed to migrate on fibronectin, a major extracellular matrix component of bone marrow[31], to a gradient of CXCL12. Individual line tracings and circular histograms (Figure 2B) show the overall speed and direction of migration, and demonstrates the variation in the rate of movement of individual cells. PECAM-1^−/−^ cells exhibited a more random migratory pattern consistent with loss of CXCR4 polarisation which has been observed in other PECAM-1^−/−^ cell types[20]. 88% of WT cells move to within a 120° arc facing the CXCL12 source, whereas only 52% of PECAM-1^−/−^ CD117^+^ cells do so. The cell traces also demonstrate that PECAM-1^−/−^ cells move relatively short distances before changing direction, suggesting that they also lack directional persistence (Figure 2C).

To determine if the reduced migration to CXCL12 in the PECAM-1^−/−^ HSCs was due to altered expression of cell surface receptors on the HSCs, key molecules involved in migration to CXCL12 and in migration on cell matrices were quantified using flow cytometry (Figure 2D). Importantly, the expression of CXCR4 and a range of integrin receptors were the same on WT and PECAM-1^−/−^ HSC, suggesting that the reduced response to CXCL12 by PECAM-1^−/−^ HSCs is an intrinsic defect, and not due lack of expression of the CXCR4 receptor. Despite the slightly increased β1 integrin expression on PECAM-1^−/−^ cells, this integrin is in the normal activation state as shown by expression of the activation epitope specific antibody 9EG7[32], [33]. Thus the defect in migration of the PECAM-1^−/−^ does not appear to be due to inappropriate activation of β1 integrins following PECAM-1 deletion.

### Normal expression of CXCL12, but increased MMP-9 expression in the bone marrow microenvironment in PECAM-1^−/−^ mice

**Figure 3:**
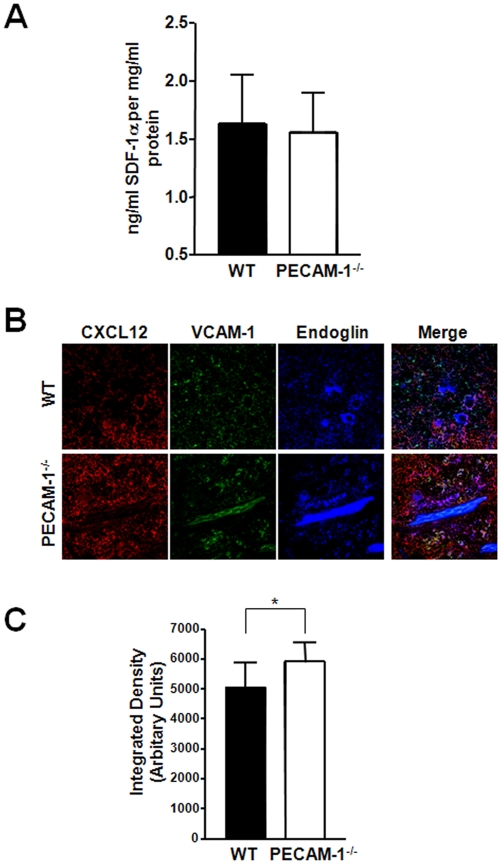
Effects of PECAM-1^−/−^ deletion on the bone marrow microenvironment. PECAM-1^−/−^ and WT littermate bone marrow was collected in PBS and analysed for factors important in HSC mobilisation. (A) PECAM-1^−/−^ bone marrow has comparable levels of CXCL12 compared to WT littermates, as measured by ELISA (n = 13). (B) Longitudinal sections of whole murine femora from matched pairs of PECAM-1^−/−^ and WT littermates were immunofluorescently stained for components of the bone marrow microenvironment; CXCL12 (red), VCAM-1 (green) and marrow sinusoidal vessels by endoglin (CD105, blue). Images are representative of at least three pairs of femurs, and images are taken at ×40 magnification. (C.) In-gel zymography demonstrated that PECAM-1^−/−^ bone marrow flushes contained higher levels of MMP-9. Pooled data from 6 litter matched pairs confirmed this to be a significant difference (data shown as the mean±S.D., *p = 0.003).

PECAM-1 is expressed not only on cells of the haematopoietic lineage but also on blood vessels in the bone marrow. This raised the possibility that factors intrinsic to bone marrow vascular cells might also contribute to the increase HSC numbers in the blood of PECAM-1^−/−^ mice. To address this question, total levels of CXCL12 present in the bone marrow were quantified by ELISA (Figure 3A). CXCL12 levels were not statistically different between PECAM-1^−/−^ and WT littermates, suggesting that the increased number of CFCs in the blood is unlikely to be due to loss of CXCL12 in the bone marrow. Levels of stem cell factor (SCF), another important factor in HSC release into the circulation[34] were also measured and these were similar between PECAM-1^−/−^ and WT mice (data not shown).

In addition, immunofluorescence microscopy was performed on cryofrozen longitudinal sections of bone marrow to see if the localization of CXCL12 was different between WT and PECAM-1^−/−^ mice. Figure 3B shows representative images of sections stained for CXCL12, using endoglin (CD105) as a vascular marker, and VCAM (CD106) as a stromal cell marker. Confocal microscopy gave similar results to the ELISA data, with no clear difference in levels or spatial localization of CXCL12. There was no significant difference in VCAM expression or localization. This implies that CXCL12 expression and localization in the bone marrow vascular niche is broadly similar, compared to WT littermates.

Functional levels of MMP-9, a protease which is important in HSC homeostasis and release from the BM[2], were quantified using in gel zymography. MMP-9 has been previously shown to be associated with PECAM-1 activation[35], and other MMPs have been found to be increased in other PECAM-1^−/−^ cell types[36]. PECAM-1^−/−^ bone marrow had significantly increased levels of MMP-9 compared to WT littermates (Figure 3C), suggesting that the loss of PECAM-1^−/−^ in the bone marrow might contribute to the defects we observed.

### HSC accumulation in the peripheral blood of PECAM-1^−/−^ mice *in vivo* is due to factors intrinsic to HSC progenitor cell migration as well as blood vessel function

**Figure 4:**
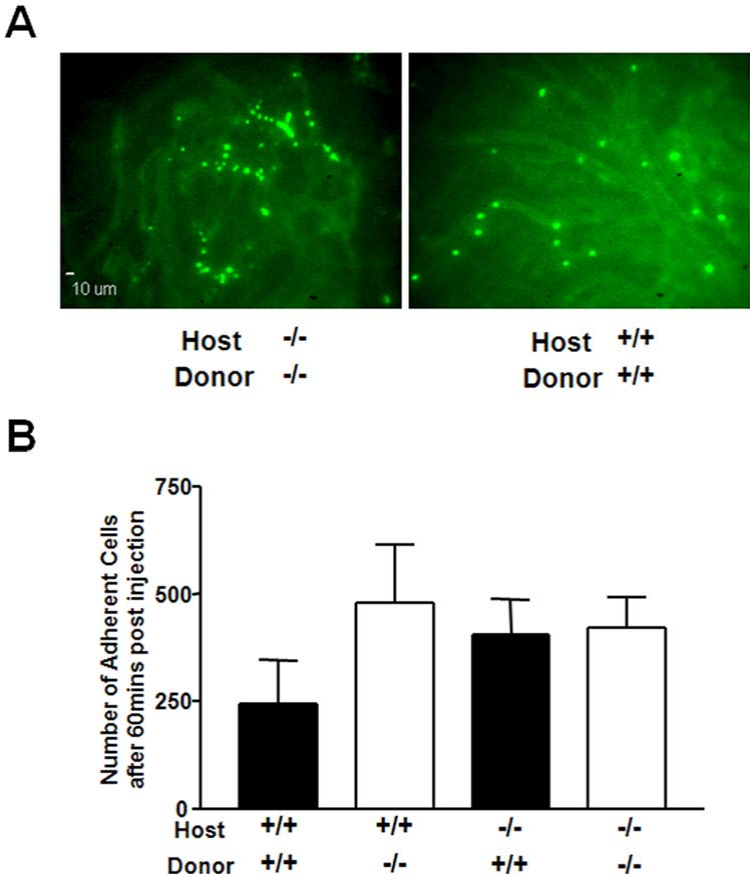
Loss of PECAM-1^−/−^ on either HSCs or endothelium affects cell adhesion to the bone marrow vasculature. CFSE labelled bone marrow cells from littermates were infused into recipient mice through a canulae in the carotid artery. Vessels in the skull were visualised with an infusion of fluorescine dye, selected fields recorded for 5 mins, and then images collected over the entire exposed vasculature 60 mins post injection. (A) Representative images of fields of view captured. (B) The number of cells adherent to the bone marrow microvessels 60 mins post injection in the exposed left frontoparietal area was quantified. Data shown is the mean±S.D of at least three independent experiments per condition, but was not statistically significant using ANOVA analysis.

In order to determine whether the increase in HSC progenitors in the blood of PECAM-1^−/−^ mice could be explained by a defect in the ability of these cells to adhere and migrate between the blood compartment and the bone marrow vasculature, we used an intra-vital skull window model to directly observe progenitor cells interacting with bone marrow blood vessels[24]. Bone marrow cells were fluorescently labelled with CFSE and injected into the carotid artery. Bone marrow blood vessels were identified by pre-administration of FITC labelled albumin (Figure 4A). Representative images show CFSE labelled cells adhering to the smaller sinusoidal vessels in the skull; regions which are thought to be similar to those in the bone marrow. The cells imaged are located on the luminal surface of the endothelial cells, having been captured and tethered from flow.

One hour post injection, the number of cells adherent to the visualised vessels was quantified (Figure 4B). Using various combinations of host animals with donor cells, we observed that both PECAM-1^−/−^ and WT cells could adhere directly to bone marrow vessels. However genetic deletion of PECAM-1 on transferred cells or recipient bone marrow lead to an increase in the number of cells accumulating on blood vessels in the bone marrow vasculature. Although not statistically significant, this suggests that deletion of PECAM-1 from either the host vasculature or donor HSC results in a delay in the ability of the bone marrow cells to cross endothelium as measured by their increased adherence to the bone marrow lumen. A similar observation was made when PECAM-1^−/−^ bone marrow cells were transferred into PECAM-1^−/−^ mice, suggesting that deletion of PECAM-1 on either the bone marrow precursor cells or the vasculature is sufficient to lead to observed phenotype, consistent with loss of homophilic interactions between PECAM-1 expressed on HSC and the bone marrow vasculature.

## Discussion

In order to move between the bone marrow and blood compartments, bone marrow progenitor cells have to migrate across vascular cells, which permit the bidirectional movement of haematopoietic cells between these two compartments. Although PECAM-1, expressed on blood and vascular cells has been shown to play an important role in regulating the movement of leucocyte subsets into peripheral tissues, it has remained unclear whether PECAM-1 plays a similar role in regulating progenitor cell migration across the bone marrow vasculature. In this study we describe a novel role for PECAM-1 in regulating progenitor cell numbers in the peripheral blood and show that part of the mechanism by which this occurs is via the effect on CXCL12 dependent HSC migration as well as an increase in MMP-9 expression upon deletion of PECAM-1 on bone marrow vascular cells.

Our data demonstrates that loss of PECAM-1 does not affect the homeostatic balance of HSCs in the bone marrow, resulting in equivalent numbers of both KSL and total mature cells of all lineages. These observations are at odds with those reported by Wu *et al* [21], who observed a statistically significant hyper cellular marrow in the PECAM-1^−/−^ mice. While we did observe a wider range of KSL numbers in the PECAM-1^−/−^ mice this was not significantly different to WT mice. Further analysis of our data showed that six pairs of mice pairs had less KSL cells defined as KO bone marrow having ≤0.9 fold of WT KSL numbers. Nine pairs had more KSL cells defined as KO bone marrow having ≥1.1 fold of WT KSL numbers. Wu *et al* [21], examined an expanded KSL population based on three pairs of mice. Several studies have reported that intrinsic natural variation between animals warrants the use of substantial animal numbers when comparing stem cell numbers between littermate pairs[37], [38]. The differences between our data and that of Wu *et al.* [21] may reflect the size of animal groups being used in each study.

Our data demonstrates that PECAM-1^−/−^ HSCs have a reduced ability to respond to CXCL12 gradients, which results physiologically in an increased number of progenitors in the peripheral circulation. CXCL12 gradients are crucially important in regulating HSC biology in both the osteoblastic and vascular niches of the bone marrow. CXCL12 produced by osteoblasts is thought to not only provide survival, adhesive and proliferation signals, but is also used in the re-homing of HSCs to the osteoblastic niche[39], [40]. G-CSF induced mobilisation of HSCs results in a dramatic reduction of CXCL12 produced by osteoblasts, thus losing the retention signal required to keep them in the bone marrow[23]. The signalling mechanisms that underpin these CXCL12 mediated effects are still to be elucidated but the Rho GTPases, Rac1 and Rac2 has been shown to be required for HSC mobilisation and homing[41].We have no direct evidence that the osteoblastic niche in the PECAM-1^−/−^ mice is abnormal, as normal levels of CXCL12 are present in the bone marrow (Figure 3A). We have however noted that the bone marrow of PECAM-1^−/−^ mice contains increased levels of MMP-9 (Figure 3C), a proteolytic enzyme which is known to inactivate CXCL12 through cleavage of its NH_2_-terminal signal sequence[42]. Thus the increase in HSC mobilisation in the resting state of the PECAM-1^−/−^ could be explained by deactivation of exogenous CXCL12 by the excess MMP-9 present in the bone marrow, or protease cleavage of other retention factors such as SCF, resulting in increased mobilization from the bone marrow under steady state conditions.

Using intra vital microscopy we have found that PECAM-1 is involved in the migration of bone marrow progenitors from the blood to the bone marrow. We observed an increase in the number of cells binding to the lumen of smaller sinusoidal vessels in the skull when PECAM-1 is deleted from either the endothelium or adoptively transferred cells. These vessels have been previously demonstrated to be physiologically similar to the bone marrow vasculature[24], and suggests that the PECAM-1^−/−^ mouse has a general defect in the ability of cells to interact with both the peripheral and bone marrow vasculature *in-vivo*. This observation provides an alternative explanation as to the increase in HSC numbers in the peripheral blood, as these cells may have a reduced ability to traffic into the bone marrow or other tissues, resulting in an accumulation in the peripheral blood. Unfortunately our intravital studies are limited to using whole bone marrow isolates, as it was not feasible to look at the trafficking of purified HSCs using this technique due to the small numbers of cells that can be isolated and enriched from the bone marrow.

In the vascular niche, CXCL12 can induce transmigration of HSCs across endothelium, in a P- and E-selectin[43] dependant process. Integrins are also important as induced deletion of either CD29 (β1 integrin), CD49d (VLA-4) or ligands such as VCAM-1 results in increased numbers of progenitor cells in the circulation[44]. The PECAM-1^−/−^ phenotype resembles elements of these deletion studies which also have increased number of circulating progenitor cells, suggesting that alternative adhesion molecules can compensate in the absence of PECAM-1 expression. We have not observed any significant differences between PECAM-1^−/−^ and WT cells in their ability to adhere or roll on selectin coated surfaces *in vitro* (data not shown). However, as reported in many other studies, challenged PECAM-1^−/−^ mice demonstrate an inflammatory response which is more aggressive and prolonged[45]–[47]. Our results suggest that part of these differences may occur because of the defect in HSC migration between the blood and BM compartments,

It is likely that the reduced ability of PECAM-1^−/−^ HSCs to migrate to CXCL12 is due to loss of an intrinsic signalling pathway mediated by the cytoplasmic domain of PECAM-1. The PECAM-1 cytoplasmic domain contains two conserved ITIM domains, and studies have demonstrated that PECAM-1 can have both inhibitory and activatory effects in various cell types[48]. It has been postulated that PECAM-1 can act as a “relay post” for modulating other surface receptors through sequestration of cytoplasmic signalling molecules such as catenins, SHP-1 and SHP-2, Src family kinases and STATs[49]–[54]. PECAM-1 activation can modulate integrin function in leucocytes, by selectively activating the small GTPase Rap1[57], which may be important for PECAM-1 mediated increase in cell migration[55], [56]. The exact signalling pathway utilised by PECAM-1 is yet to be fully described in any cell type studied, including HSCs. However, signalling intermediates known to be activated by PECAM-1, such as Rap1, have already been reported to modulate integrin function in HSCs[41].

Our study has revealed a novel role for PECAM-1 in the biology of HSCs in the bone marrow and their ability to respond to a CXCL12 gradient, which we propose results in an increased number of progenitor cells in the peripheral circulation. Using in-vivo imaging, we have observed a reduced ability of cells to migrate across bone marrow vascular endothelium upon PECAM-1 deletion. We could find no significant difference in numbers of HSCs or cells derived from them in the bone marrow suggesting that loss of PECAM-1 does not affect hematopoietic homeostatic mechanisms in the bone marrow compartment in the resting state. We have however found higher levels of MMP-9 in the bone marrow of PECAM-1^−/−^ mice suggesting that the vascular niche in the bone marrow is abnormal in PECAM-1^−/−^ mice. These findings further support a role for PECAM-1 in modulating cellular migration and adhesion and extend the vascular compartments regulated by PECAM-1 to include the bone marrow vasculature niche.
